# Changes in Oral Function and Quality of Life in Tongue Cancer Patients Based on Resected Area

**DOI:** 10.31557/APJCP.2021.22.8.2549

**Published:** 2021-08

**Authors:** Yoshiaki Ihara, Yuichi Tashimo, Shinji Nozue, Yoshiki Iizumi, Yuma Fukunishi, Yoshiro Saito, Toshikazu Shimane, Koji Takahashi

**Affiliations:** 1 *Division of Oral Rehabilitation Medicine, Department of Special Needs Dentistry, School of Dentistry, Showa University, Tokyo, Japan. *; 2 *Head and Neck Oncology Center, Showa University Hospital, Tokyo, Japan. *

**Keywords:** Oral function, quality of life, tongue cancer, glossectomy

## Abstract

**Objective:**

Treatment of tongue cancer caused oral morbidities such as oral dryness, and dysphagia. The purpose of this study is to examine the time course of oral function and QOL based on resected area for patients after tongue cancer resection.

**Methods:**

31 patients who underwent tongue cancer resection at the Showa University Head and Neck Oncology Center. The participants were divided into two groups; 24 participants in partial/hemi glossectomy group (PG), and seven in subtotal/total glossectomy group (TG). Participants were evaluated swallowing function (FOIS and MASA-C), tongue pressure (TP: kPa), BMI, whole body muscle mass (kg), and QOL evaluation (EORTC QLQ-C30, H & N35). Participants were measured at baseline (before surgical treatment), 1, 3, and 6 months after surgical treatment (1M, 3M, and 6M).

**Results:**

At baseline, tongue pressure and FOIS score of PG were significant higher than that of TG. At 1M, TP, MASA-C, and FOIS score of PG were significant higher than that of TG. At 3M, TP, MASA-C, and FOIS score of PG were significant higher than that of TG. At 6M, TP and MASA-C were significantly higher than that of TG. QOL measurements did not noted any significant difference between groups before 6M. At 6M, Some QOL measurements of TG related tongue function (Swallowing, Senses, Speech, Social contact) were significantly lower than PG.

**Conclusions:**

The resected area had significant effects on oral morbidities and feeding function. It is necessary to develop more effective rehabilitation methods to improve patients QOL who had functional impairment remained.

## Introduction

Treatment of tongue cancer (TC) contributes significantly to oral dysfunction (Raj et al., 2019). Oral dysfunction is often long lasting, given that TC prevalence is increasing in younger patients and the survival rates are increasing (Young et al., 2015). Previous studies have reported that the frequency of oral dysfunction, including difficulty in swallowing (dysphagia) and speech difficulty, caused by TC treatment depends on the resected area of the tongue (Yasuo, 1992). Dysphagia has been reported in over 76% of head and neck cancer (HNC) patients treated with concurrent chemotherapy. It decreases the patient’s quality of life (QOL) following HNC treatment (Greco et al., 2018). QOL is considered to be an important factor in both treatment decision and outcome evaluation (Anuradha et al., 2013; Blazeby et al., 1995; Goncalves and Rocha, 2012; Maciejewski et al., 2010). It is necessary for multidirectional analysis and for the appropriate evaluation of treatment results. The result of HNC treatment should be evaluated according to both QOL and post-treatment functional outcomes (Guenzel et al., 2018). However, only a few studies have conducted a multidirectional analysis that includes QOL by focusing on change over time, following HNC treatment. Further, a majority of previous studies have focused on HNC patients who received chemoradiation therapy (Greco et al., 2018; van den Berg et al., 2014; Ruten et al., 2011; Ihara et al., 2018; Hutcheson et al., 2012). In contrast, a few studies have focused on the change in QOL for HNC patients who received surgical treatment (Tashimo et al., 2019). In HNC patients who received chemoradiation therapy, some studies have reported that the QOL returned to baseline a year after treatment (Gritz et al., 1999; Marzouki et al., 2018). However, it has been reported that the QOL of HNC patients who received surgical treatment did not return to baseline after treatment (Tashimo et al., 2019). The longitudinal change in QOL in HNC patients who underwent surgery is still unclear. Moreover, the relationship between the resected area and decrease in oral function and QOL is also unclear. Thus, the purpose of this study was to examine the differences in oral function and QOL in patients after TC resection, based on the resected area.

## Materials and Methods

*Patients*

This study included TC patients who were scheduled for surgical treatment at the Head and Neck Oncology Center, Showa University Hospital and were then referred to the Department of Special Needs Dentistry, Division of Oral Rehabilitation Medicine, Showa University Dental Hospital for rehabilitation. The participants were divided into two groups: partial glossectomy or hemi-glossectomy (PG) group and subtotal/total glossectomy (TG) group. The exclusion criteria were: (1) age<20 years, (2) inability to follow instructions, (3) other malignant tumors, (4) severe diseases that could influence the evaluation, (5) incomplete measurement data, and (6) provision of additional treatment (e.g., chemotherapy, radiation therapy) due to the recurrence of the cancer. Patients were examined at baseline (BL; before surgical treatment), 1 month (1M; 1 month after surgical treatment), 3 months (3M; 3 months after surgical treatment), and 6 months (6M; 6 months after surgical treatment). This study was approved by the Ethics Committee of Showa University School of Medicine (Approval no. 2355), and all participants signed an approved informed consent form before participating in the study. All procedures were performed according to the World Medical Association Declaration of Helsinki (version, 2002).

*Assessments*

All measurements were performed by dentists from the Department of Special Needs Dentistry, Division of Oral Rehabilitation Medicine, Showa University Dental Hospital. The primary tumor stage, TNM classification, method of surgical operation, use of a palatal augmentation prosthesis, and medical history were collected from the medical records. The patient’s weight, body mass index (BMI), whole body soft lean mass (SLM), and skeletal muscle mass (SMM) were evaluated as muscle mass-related measurements. Lip closure force (LC) and tongue pressure (TP) were evaluated as oral function measurements. Feeding function was evaluated using the Mann Assessment of Swallowing Ability–Cancer version (MASA-C) and Functional Oral Intake Scale (FOIS), while QOL was assessed using the European Organization for Research and Treatment of Cancer (EORTC) QOL Questionnaires QLQ-C30 and QLQ-H&N 35.

*Muscle mass-related measurements*

SLM and SMM were measured using Inbody S20 (BioSpace, Seoul, Korea), which can evaluate the patient’s SLM and SMM in a supine position. The patient was placed in a supine position on the examination table, with four electrodes on the first and third fingers and four points on the left and right ankles, eight contact-type electrodes in total (Okamoto et al., 2006). The patient’s weight was measured, and BMI was calculated at each time point (BL, 1M, 3M, and 6M).

*Oral function measurements*

LC was measured five times using a lip force measuring device (Lip de Cum model LDC-110R, Cosmo-Instruments Co, Ltd, Tokyo, Japan). The average score of the five measurements was calculated as the LC score (Naoko et al., 2015).

TP was evaluated using the JMS tongue pressure measuring device (JMS Co. Ltd., Hiroshima, Japan). A balloon-shaped intraoral probe was placed behind the upper front teeth. The patients were instructed to push the probe against the hard palate using the tongue with maximum force for five seconds, and changes in air pressure inside the probe were measured. The measurements were performed 10 times, and the average score was calculated as the final TP score (Hasegawa et al., 2017). Between each trial, there was an interval of at least one minute to recover from fatigue.

*Feeding function*

Swallowing function was evaluated using the MASA-C (Carnaby and Crary, 2014). MASA-C has been validated for use in the HNC population and demonstrates strong sensitivity, specificity, and positive predictive value for the identification of dysphagia. The total maximum score that can be obtained from MASA-C is 200 points. A cut-off score of 185 determines the presence of dysphagia in the HNC population.

The FOIS was used as a measure of functional eating status (Crary et al., 2005). It is a valid and reliable tool used to document functional eating abilities. A 7-point ordinal scale describes the functional oral intake of patients with dysphagia.

*QOL measurements*

QOL was assessed using the Japanese version of EORTC QLQ-C30 version 3.0 and QLQ-H&N35 questionnaires. The scores were calculated according to the EORTC scoring manual (Aaronson et al., 1993; Fayers et al., 2001). The QLQ-C30 is composed of both multi-item scales and single-item measures, and includes five functional scales, three symptom scales, a global health status/QOL scale, and six single items. Each of the multi-item scales includes a different set of items; no item occurs in more than one scale. All of the scales and single-item measures range in score from 0 to 100. A high scale score represents a higher response level. Thus, a high score for a functional scale represents a high/healthy level of functioning, a high score for the global health status/QOL represents a high QOL, but a high score for a symptom scale/item represents a high level of symptomatology/problems. EORTC QLQ-H&N35 incorporates seven multi-item scales that assess pain, swallowing, senses (taste and smell), speech, social eating, social contact, and sexuality. It also includes 11 single items. All the scales and single-item measures range in score from 0 to 100. For all items and scales, high scores indicate more problems.

*Rehabilitation*

All patients enrolled in this study received indirect or direct training, as needed. After the pain in the surgical region had subsided, patients underwent rehabilitation such as massage for surgical scarring, oral motor exercise including lingual exercise, and direct training, according to their requirement. Moreover, some patients underwent swallowing rehabilitation and used a palatal augmentation prosthesis (PAP) to improve bolus transportation, pharyngeal swallowing pressure, and clearance of the oral and pharyngeal residue.

*Statistical analysis*

Bivariate analyses with t-tests, *χ*^2^ and Fisher’s exact tests were used to compare the two groups for age, gender, muscle mass-related measurements, oral function measurements, feeding function, and QOL measurements. Paired t-tests and the sign test were used to detect changes within groups from baseline to six months. Statistical analyses were performed using IBM SPSS version 25.0 (IBM, New York, USA). All p values were two-sided, and p<0.05 was considered significant.

This study was approved by the Ethics Committee of Showa University School of Medicine (Approval no. 2355), and all participants signed an approved informed consent form before participating in the study. All procedures were performed according to the World Medical Association Declaration of Helsinki (version 2002).

## Results

*Patients*

A total of 31 patients (19 men and 12 women) were included in this study. The mean patient age was 62.90 years (standard deviation [SD]: 13.69 years). The participants were divided into PG and TG groups. The patients’ characteristics are described in detail in Table 1.

*Muscle mass-related measurements*

BMI: There was no significant difference in BMI between PG and TG groups at each time point [(BL: PG- mean 24.44, SD = 3.44 kg/m^2^; TG- mean 24.44, SD = 4.07 kg/m^2^) (1M: PG- mean 23.59, SD = 3.50 kg/m^2^; TG- mean 23.00, SD = 2.97 kg/m^2^) (3M: PG- mean 23.96, SD = 3.55 kg/m^2^; TG- mean 21.97, SD = 3.07 kg/m^2^) (6M: PG- mean 24.15, SD = 3.60 kg/m^2^; TG- mean 22.5, SD = 1.83 kg/m^2^)].

SLM: There was no significant difference in SLM score between PG and TG groups at each time point [(BL: PG- mean 39.82, SD = 10.76 kg; TG- mean 44.63, SD = 10.62 kg) (1M: PG- mean 40.86, SD = 9.40 kg; TG- mean 45.20, SD = 6.67 kg) (3M: PG- mean 40.76, SD = 9.83 kg; TG- mean 44.27, SD = 10.21 kg) (6M: PG; mean 45.58, SD = 10.10 kg; TG- mean 47.98, SD = 5.42 kg)].

SMM: There was no significant difference in SMM score between PG and TG groups at each time point [(BL: PG- mean 23.01, SD = 6.94 kg; TG- mean 25.85, SD = 6.78 kg) (1M: PG- mean 23.46, SD = 5.99 kg; TG- mean 25.92, SD = 4.08 kg) (3M: PG- mean 23.45, SD = 6.27 kg; TG- mean 25.32, SD = 6.35 kg) (6M: PG- mean 26.68, SD = 6.59 kg; TG- mean 27.74, SD = 3.41 kg)].

*Oral function measurements*

**Figure 1:**
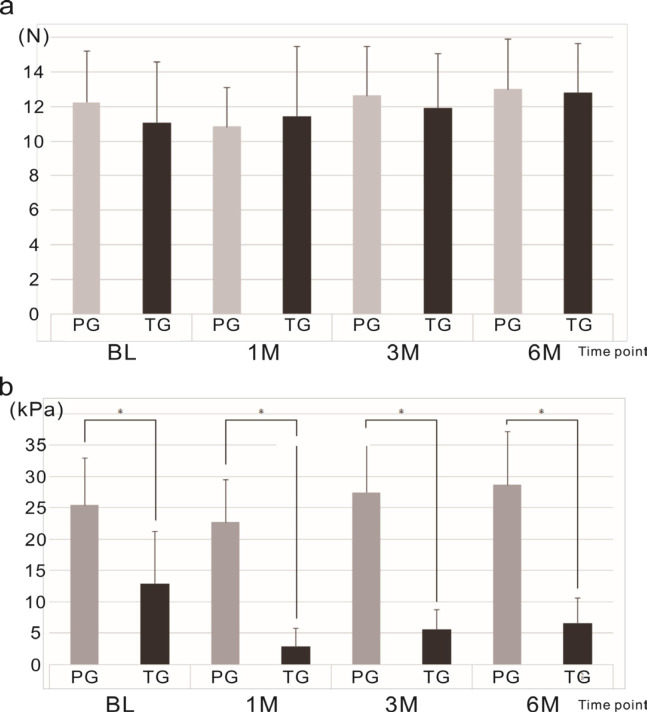
Oral Function Outcomes. a, Mean Lip closure force (LC) at baseline, 1 month, 3 months, and 6 months post-treatment; b, Mean tongue pressure (TP) scores at baseline, 1 month, 3 months, and 6 months post treatment. *p<0.05, **p<0.01

LC: There was no significant difference in LC score between PG and TG groups at each time point [(BL: PG- mean 12.24, SD = 2.96 N; TG- mean 11.09, SD = 3.48 N) (1M: PG- mean 10.84, SD = 2.26 N; TG- mean 11.46, SD = 4.02 N) (3M: PG- mean 12.65, SD = 2.85 N; TG- mean 11.89, SD = 3.16 N) (6M: PG- mean 13.01, SD = 2.91 N; TG- mean 12.81, SD = 2.83 N)] (Figure 1a).

TP: The mean TP score for the PG group was significantly higher than that for the TG group at all measurement points [(BL: PG- mean 25.43, SD = 7.58 kPa; TG- mean 12.89, SD = 8.38 kPa; p<0.01) (1M: PG- mean 22.75, SD = 6.70 kPa; TG- mean 2.87, SD = 2.87 kPa; p<0.01) (3M: PG- mean 27.50, SD = 7.96 kPa; TG- mean 5.64, SD = 3.06 kPa; p<0.01) (6M: PG- mean 28.67, SD = 8.51 kPa; TG- mean 6.56, SD = 4.01 kPa; p<0.01)] (Figure 1b).

*Feeding function*

**Figure 2:**
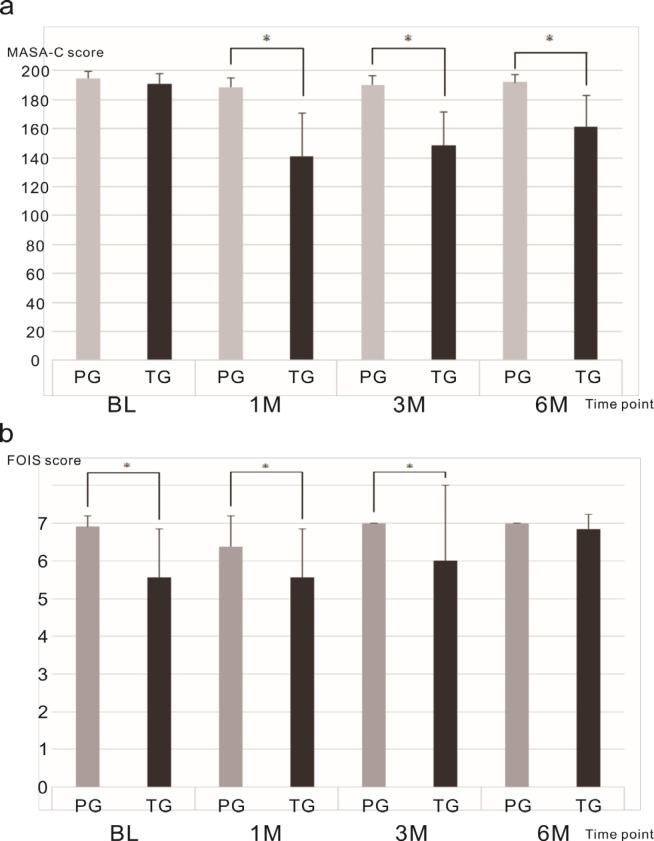
Feeding Function Outcomes. a, Mean Mann Assessment of Swallowing Ability–Cancer version (MASA-C) scores at baseline, 1 month, 3 months, and 6 months post treatment; b, Mean Functional Oral Intake Scale (FOIS) ratings at baseline, 1 month, 3 months, and 6 months post treatment. *p<0.05, **p<0.01

MASA-C: At BL, there was no significant difference in the MASA-C score between the PG (mean 194.72, SD = 4.91) and TG (mean 191.00, SD = 6.78) groups. At 1M, the mean MASA-C score of the PG group was significantly higher than that of the TG group (PG- mean 188.80, SD = 6.61; TG- mean 140.75, SD = 29.81; p<0.01). At 3M, the mean MASA-C score of the PG group was significantly higher than that of the TG group (PG- mean 190.47, SD = 6.41; TG- mean 148.75, SD = 22.65; p<0.01). At 6M, the mean MASA-C score of the PG group was significantly higher than that of the TG group (PG- mean 192.22, SD =5.21; TG- mean 161.33, SD =21.42, p<0.01) (Figure 2a).

FOIS: At BL, the mean FOIS score of the PG group (mean: 6.91, SD = 0.28) was significantly higher than that of the TG group (mean: 5.57, SD = 1.27; p<0.01). At 1M, the mean FOIS score of the PG group (mean: 6.38, SD = 0.82) was significantly higher than that of the TG group (mean: 5.57, SD = 1.27; p<0.01). At 3M, all the PG patients scored 7, and the mean FOIS score of the TG group (mean: 6.00, SD = 2.00) was significantly lower than that of the PG group (p<0.01). At 6M, all the PG patients scored 7, and there was no significant difference between the mean FOIS score of PG and TG groups (mean: 6.83, SD = 0.41; p=0.06) (Figure 2b).

*QOL measurements*

Table 2 presents the results of EORTC QLQ-C30. There was no significant difference between PG and TG groups at any point of measurement. Table 3 presents the results of EORTC QLQ-H&N35. There was no significant difference between the PG and TG groups at BL, 1M, or 3M. However, at 6M, significant differences were noted between the PG and TG groups. In QLQ-C30, pain in the PG group was significantly lower than that in the TG group (PG- mean 0, SD = 0; TG- mean 19.45, SD = 24.53; p = 0.01). In QLQ-H&N35, swallowing, senses, speech, social contact, and mouth opening in the PG group were significantly lower than that in the TG group. (swallowing: PG- mean 3.85, SD = 6.47; TG- mean 16.67, SD = 9.13; p = 0.008, senses: PG- mean 0, SD = 0; TG- mean 22.22, SD = 32.77; p = 0.012, speech: PG- mean 3.03, SD = 7.18; TG- mean 27.31, SD = 16.33; p = 0.005, social contact: PG- mean 0, SD = 0; TG- mean 25.56, SD = 33.31; p = 0.003, mouth opening: PG- mean 0, SD = 0; TG- mean 16.67, SD = 27.89; p = 0.048).

**Table 1:** Characteristics of Patients

	Partial glossectomy or hemi-glossectomy group: (PG), n ±SD	Subtotal/total glossectomy group: (TG), n, ±SD
Gender (Male: Female)	13:11	6:01
Age in years, Mean (SD)	64.04±14.53	59.00 ±10.26
Weight in Kg, Mean (SD)	64.51 ±13.47	65.50 ±15.57
Tumor Stage		
TisN0	1	0
T1N0	10	0
T1N1	1	1
T2N0	10	0
T2N1	1	0
T3N0	1	1
T3N1	0	1
T4aN1	0	2
T4aN2	0	1
T4bN0	0	1
Reconstruction		
Forearm flap	2	1
Rectus abdominis myocutaneous flap	0	1
Pectoralis major musculocutaneous flap	0	3
anterolateral thigh flap	2	2

**Table 2:** Result of European Organization for Research and Treatment of Cancer Quality of Life Questionnaire-C30

		BL^a^	^1^M^b^	^3^M^c^	^6^M^d^
		Mean±SD	Mean±SD	Mean±SD	Mean±SD
Global health status	PG^e^	59.72±27.22	70.29±23.28	72.10±21.85	78.79±14.61
	TG^f^	63.57±32.52	61.90±23.00	73.81±21.21	65.28±13.35
Physical functioning	PG	95.56±7.53	91.59±10.29	89.28±16.30	96.97±6.23
	TG	96.19±6.50	88.57±13.72	93.33±6.67	92.22±6.55
Role functioning	PG	97.22±10.62	88.41±15.43	88.10±15.85	98.48±5.03
	TG	80.95±37.80	88.10±88.41	92.03±20.02	97.22±6.81
Emotional functioning	PG	81.25±22.69	88.77±15.81	89.49±12.62	93.94±8.41
	TG	82.14±24.26	75.00±31.55	92.86±10.13	84.72±18.57
Cognitive functioning	PG	89.58±12.83	87.68±20.24	86.23±15.61	95.45±7.79
	TG	78.57±35.63	90.48±13.11	92.86±8.91	88.89±13.61
Social functioning	PG	92.36±14.73	95.45±9.18	90.58±15.75	98.48±5.03
	TG	76.19±35.82	78.57±20.89	90.48±16.26	88.89±13.61
Fatigue	PG	16.20±13.89	21.25±18.01	19.32±22.02	11.11±11.11
	TG	19.05±17.82	19.05±8.40	15.87±16.80	24.07±19.14
Nausea and vomiting	PG	2.08±7.47	5.07±20.98	2.90±9.60	0±0
	TG	2.38±6.30	0±0	0±0	0±0
Pain	PG	15.28±18.33	17.39±18.45	13.04±19.43	0±0
	TG	28.57±39.33	9.52±13.11	11.90±15.85	19.45±24.53
Dyspnea	PG	2.78±9.41	7.25±17.28	10.14±23.43	3.03±10.05
	TG	0±0	4.76±12.60	4.76±12.60	16.67±27.89
Sleep	PG	18.05±19.61	18.18±22.37	16.67±26.73	6.06±13.48
	TG	19.05±26.23	28.57±30.00	14.28±17.82	5.56±13.61
Appetite loss	PG	9.52±16.26	11.59±19.09	5.80±12.92	3.03±10.05
	TG	8.33±14.74	19.05±17.82	0±0	0±0
Constipation	PG	6.94±13.83	18.84±22.08	8.69±14.96	3.03±10.05
	TG	28.57±40.50	19.05±17.82	4.76±12.60	16.67±18.26
Diarrhea	PG	2.78±9.41	7.25±14.06	7.25±14.06	6.06±13.48
	TG	4.76±12.60	4.76±12.60	14.28±17.82	5.56±13.61
Financial difficulties	PG	11.11±25.38	4.35±11.48	10.14±25.49	9.09±30.15
	TG	14.29±26.23	19.05±26.23	9.52±16.26	5.56±13.61

^a^BL, Baseline; ^b^1M, 1 month after surgical treatment; ^c^3M, 3 months after surgical treatment; ^d^6M, 6 months after surgical treatment; ^e^PG, partial glossectomy or hemi-glossectomy; ^f^TG, subtotal/total glossectomy.

**Table 3:** Result of European Organization for Research and Treatment of Cancer Quality of Life Questionnaire-H&N35

		BL^a^	^1^M^b^	^3^M^c^	^6^M^d^
		Mean±SD	Mean±SD	Mean±SD	Mean±SD
Pain	PG^e^	17.01±13.57	13.41±15.64	4.76±9.45	5.30±10.05
	TG^f^	28.57±31.86	11.90±15.85	9.42±11.87	13.89±16.39
Swallowing	PG	19.05±27.78	17.39±20.24	15.22±23.92	3.85±6.47**
	TG	12.85±18.39	23.81±27.40	14.68±13.20	16.67±9.13
Senses problems	PG	4.86±10.40	7.25±15.75	6.52±13.98	0±0*
	TG	2.38±6.30	7.14±13.11	4.76±12.60	22.22±32.77
Speech problems	PG	3.70±8.50	22.95±22.99	15.46±21.64	3.03±7.18**
	TG	12.70±25.20	33.33±29.40	15.87±17.98	27.31±16.33
Trouble with social eating	PG	20.14±18.21	27.54±21.97	24.28±21.75	13.63±11.35
	TG	23.81±26.10	32.14±23.29	19.05±15.75	34.72±23.82
Trouble with social contact	PG	4.72±10.67	18.94±26.27	15.65±26.26	0±0**
	TG	13.33±25.53	24.76±20.26	10.48±12.09	25.56±33.31
Less sexuality	PG	13.89±23.91	17.39±25.86	16.67±25.13	4.55±10.78*
	TG	30.95±39.00	9.52±16.26	23.81±25.20	22.22±17.21
Teeth	PG	9.72±18.33	8.70±18.03	10.14±15.68	0±0
	TG	4.76±12.60	14.28±17.82	4.76±12.60	5.56±13.61
Opening mouth	PG	16.67±29.49	15.94±24.35	11.59±25.84	0±0*
	TG	14.29±26.23	19.05±26.23	14.28±17.82	16.67±27.89
Dry mouth	PG	12.50±16.48	21.74±23.80	17.39±24.35	12.12±22.47
	TG	23.81±31.71	19.05±26.23	9.52±16.26	11.11±17.21
Sticky saliva	PG	19.44±27.66	20.29±26.09	11.59±16.23	9.09±21.56
	TG	28.57±40.50	14.29±26.23	14.29±26.23	38.89±28.97
Coughing	PG	5.56±12.69	11.59±16.23	4.35±11.48	6.06±13.48
	TG	14.28±17.82	9.52±16.26	14.28±17.82	11.11±27.22
Felt ill	PG	18.06±27.77	14.49±38.10	8.70±18.03	6.06±13.48
	TG	19.05±32.53	40.50±16.89	9.52±16.26	16.67±27.89
Pain killers	PG	8.33±14.74	10.14±15.68	2.90±9.60	3.03±10.05
	TG	19.05±17.82	0±0	0±0	0±0
Nutritional supplements	PG	5.55±12.69	2.90±9.60	1.45±6.95	6.06±13.48
	TG	0±0	9.52±16.26	4.76±12.60	5.56±13.61
Feeding tube	PG	0±0	1.45±6.95	2.90±9.60	0±0
	TG	0±0	4.76±12.60	0±0	5.56±13.61
Weight loss	PG	6.94±13.83	5.80±12.92	1.45±6.95	3.03±10.05
	TG	14.28±17.82	14.28±17.82	4.76±12.60	0±0
Weight gain	PG	5.56±12.69	7.25±14.06	7.25±14.06	15.15±17.41
	TG	0±0	14.28±17.82	19.05±17.82	16.67±18.26

^a^BL, Baseline; ^b^1M, 1 month after surgical treatment; ^c^3M, 3 months after surgical treatment; ^d^6M, 6 months after surgical treatment; ^e^PG, partial glossectomy or hemi-glossectomy; ^f^TG, subtotal/total glossectomy. *p<0.05, **p<0.01.

## Discussion

The results of this study indicated that the area of the resected tongue had significant effects on tongue pressure (TP) and feeding function. In contrast, it did not indicate any effect on muscle mass-related measurements. QOL measurements did not demonstrate any significant difference before 6M. However, some QOL scores showed significant differences between PG and TG groups at 6M.

In a previous study, it was reported that the reduction in TP depended on cancer stage, radiotherapy, and reconstruction (Hasegawa et al., 2017). Patients who underwent minimal glossectomy, near-half partial glossectomy of the mobile tongue, and subtotal glossectomy demonstrated 6%, 59.6%, and 84.7% reduction in TP, respectively (Hasegawa et al., 2017). In the present study, patients underwent subtotal/total glossectomy, indicating almost 80% reduction at 1M. However, it recovered to about 55% reduction from BL. In contrast, in patients who underwent partial glossectomy or hemi-glossectomy, TP levels almost similar to that at BL were noted. In this study, all patients received indirect and direct training, as needed. It has been reported that 9 weeks of oral motor exercise including lingual exercise increases lingual strength (Son et al., 2015). This might be the cause of the recovery of TP in both groups.

Before treatment, there was no significant difference in the MASA-C scores between the PG and TG groups. Only one patient demonstrated dysphagia (MASA-C score<185). This patient claimed an inability to eat a normal diet because of pain. However, after treatment, all TG patients indicated dysphagia. Thus, regardless of the size of the cancer, its presence causes dysphagia; however, no PG patients reported dysphagia. This suggested that patients with greater than half glossectomy had a higher incidence of dysphagia, with or without reconstruction. A previous study reported that the extent of tumor resection and lymph node metastasis affected swallowing in TC patients (Son et al., 2014). Another study reported that patients with tongue resection greater than 50% and advanced tumor stage were at a high risk for aspiration (Huang et al., 2016). Dysphagia is among the most prevalent and debilitating symptoms resulting from HNC treatment. It has been reported that different mechanisms may contribute to the development and maintenance of dysphagia during HNC treatment (Ihara et al., 2018). Studies have investigated the risk of aspiration in patients with tongue resection due to cancer. In this study, we used MASA-C to detect dysphagia. All TG patients reported dysphagia after treatment, regardless of the presence/absence of aspiration. Patients had undergone swallowing rehabilitation and used PAP, which resulted in improved bolus transportation, pharyngeal swallowing pressure, and clearance of the oral and pharyngeal residue (Ohno et al., 2017). This could be why they were able to eat a normal diet despite dysphagia (FOIS score 7). A variety of assessment tools have been developed to measure the severity of dysphagia and the level of functional eating abilities are not the same (Kunieda et al., 2013).

In this study, there were no significant differences between the QOL measurements of the two groups before 6M. However, the patient’s QOL dropped significantly after treatment. Some scales did not return to BL at 6M. Previous studies in HNC patients utilizing radiotherapy have also reported that the patient’s QOL did not return to baseline after treatment (Marzouki et al., 2018; Loorents et al., 2016). However, a previous study reported that the QOL of HNC patients returned to baseline a year after treatment; in these patients, dysfunctional eating, diet, and speech persisted, but the changes were not statistically significant (Tashimo et al., 2019). The same tendency was noted in the present study at 6M. Some QOL measurements in the TG group related to tongue function (swallowing, senses, speech, and social contact) were significantly lower than that in the PG group. Before 6M, the effect of surgery on the healing period of the wound was evident in patients of both groups, and rehabilitation could improve the QOL in both groups. The patients enrolled in this study underwent swallowing rehabilitation, including direct/indirect training, and utilized PAP. This might have contributed to the prevention of the decline in QOL. In stroke patients also, swallowing rehabilitation is reported to affect QOL (Bahceci et al., 2017). By initiating rehabilitation at an early stage, tongue function and QOL measurements in the PG group recovered at 3M. However, the results of this study showed that patients who underwent surgical resection of a greater part such as subtotal/total glossectomy showed remnant functional impairment, indicated by a lower QOL score at 6M. This result suggested that it is necessary to develop more effective rehabilitation methods to improve the QOL of patients with residual functional impairment.

It has been reported that neck dissection has a significant effect on swallowing function (Kazuo et al., 2007). If the pharyngeal branch of the vagus nerve is injured during neck dissection, the ability to clear the pharynx is reduced. In many cases of oral cancer, reconstruction of the pharynx results in a limitation of laryngeal elevation or a delay in the swallowing reflex, thereby causing aspiration. In this study, two PG patients and all TG patients underwent neck dissection. This might have had a significant effect on the result of MASA-C. The two PG patients who underwent neck dissection did not indicate dysphagia on MASA-C. This could be because the resected area of PG was small enough to preserve tongue function. In previous studies, patients with partial glossectomy did not show dysphagia because their tongue function was preserved even after treatment (Hasegawa et al., 2017; Aki et al., 2006). It was thought that tongue function could have masked the effects of cervical dissection in PG. However, tongue function in TG was not enough to mask the effects of cervical dissection.

*Study limitation*

This prospective cohort study was limited to a small sample size with further reduction in cases due to subject withdrawal during the course of the study. Patient drop-out during a prospective HNC study because of the recurrence of cancer, change of residence, etc. is not unusual (Rademaker et al., 2003; Shinn et al., 2013). However, it may introduce a degree of subject bias into the overall results. Furthermore, additional variables such as the type, amount, and duration of medications (specifically pain management medications) may provide an insight into the observed results. Thus, to better clarify the changes in QOL reported in the current study, future studies should incorporate larger samples, follow patients for a longer post-treatment interval, and consider additional variables that have the potential to affect the patients’ QOL.

In conclusion, the size of resection had significant effects on oral morbidities. However, significant effects on the participant’s QOL were not demonstrated before 6M. At 6M, some QOL measurements in the TG group related to tongue function (swallowing, senses, speech, and social contact) were significantly lower than that in the PG group. The results of this study suggested that appropriate rehabilitation might help to improve the patients’ QOL. However, it is necessary to develop more effective rehabilitation methods to improve the QOL in patients with residual functional impairment.

## Author Contribution Statement

Yoshiaki Ihara; research planning, paper writing. Yuichi Tashimo, Shinji NOzue, Yoshiki Iizumi, Yuma Hukunishi, Yoshiro Saito; data collection. Toshikazu Shimane, Koji Takahashi; study design. 
